# A Randomized, Placebo-Controlled Trial Evaluating Multi-Species Synbiotic Supplementation for Bloating, Gas, and Abdominal Discomfort

**DOI:** 10.3390/nu18020255

**Published:** 2026-01-14

**Authors:** Jessica R. Allegretti, Zain Kassam, Colleen R. Kelly, Ari Grinspan, Najwa El-Nachef, Courtney Van Den Elzen, Ralf Jäger, Paul Feuerstadt

**Affiliations:** 1 Division of Gastroenterology, Hepatology, and Endoscopy, Brigham and Women’s Hospital, Harvard Medical School, Boston, MA 02115, USA; 2 Seed Health, Inc., Venice, CA 90291, USA; 3 The Henry D. Janowitz Division of Gastroenterology, Icahn School of Medicine at Mount Sinai, New York, NY 10029, USA; 4 Division of Gastroenterology, University of California, San Francisco, CA 94143, USA; 5 Division of Gastroenterology, Henry Ford Health System, Detroit, MI 48202, USA; 6 Increnovo LLC, Whitefish Bay, WI 53217, USA; 7 Department of Digestive Diseases, Yale University School of Medicine, New Haven, CT 06510, USA; 8 Department of Gastroenterology, PACT Gastroenterology Center, Hamden, CT 06518, USA

**Keywords:** probiotic, prebiotic, synbiotic, bloating, gas, abdominal pain, constipation, regularity, GI quality-of-life, microbiome

## Abstract

**Background:** Bloating, gas, and abdominal discomfort are common in healthy individuals but lack effective interventions. Probiotics can alleviate some gastrointestinal (GI) symptoms; however, evidence for their impact on bloating, gas and abdominal discomfort in otherwise healthy populations remains limited. Mechanistic studies suggest that synbiotics may influence the underlying mechanisms of bloating, including increased gas production, impaired gut motility, and visceral hypersensitivity, but there is a paucity of data from large trials evaluating clinical outcomes. Accordingly, we evaluated the effects of a multi-species synbiotic on GI symptoms. **Methods**: In a randomized, double-blind, placebo-controlled, decentralized trial, participants (*n* = 350) with self-reported bloating/indigestion received either a multi-species synbiotic (53.6 billion AFU multi-species probiotic and 400 mg pomegranate extract; DS-01) or placebo daily for 6 weeks. Outcomes included GI quality-of-life (DQLQ), bloating and gas (PROMIS-GI 13a), abdominal discomfort (PROMIS-GI 5a), constipation, regularity, mood-related symptoms, and safety. **Results**: The multi-species synbiotic improved GI quality-of-life compared to placebo (0.80 vs. 1.20; *p* < 0.05) at Week 6. Bloating and gas were reduced in the synbiotic arm compared to placebo (16.0 vs. 21.0; *p* < 0.01), with more participants reporting never/rarely bloating (72.3% vs. 55.9%; *p* < 0.001). Abdominal discomfort also decreased (8.0 vs. 10.0; *p* < 0.01). Additionally, there was a statistically significant improvement in constipation symptoms and regularity in the synbiotic arm relative to placebo. **Conclusions**: Daily supplementation with this multi-species synbiotic significantly improved GI quality-of-life, bloating, gas, abdominal discomfort, and bowel habits. This is the first synbiotic to demonstrate meaningful improvements in bloating and gas in a generally healthy, diverse, real-world population.

## 1. Introduction

Bloating and abdominal discomfort represent common clinical concerns in the general population. Nearly one in seven Americans experience bloating on a weekly basis, and international data from 26 countries suggest an even higher global prevalence [1,2]. Excess intestinal gas and abdominal discomfort/pain are key symptoms linked to bloating severity, with up to 89% of adults in the general population reporting gas-related symptoms within a 24 h period [2,3]. The clinical impact is meaningful, as bloating and gas-related symptoms are associated with reduced quality-of-life and higher levels of stress and anxiety [3]. Despite this burden, effective interventions are limited, and available solutions lack data.

The biology of bloating is not fully understood and likely reflects multifactorial pathophysiology, with symptoms in different individuals driven by distinct mechanisms-of-action. Recently, the gut microbiome has emerged as a key multi-modal driver, exerting effects on intestinal gas production, gastrointestinal (GI) motility, and visceral hypersensitivity. First, certain microbes increase intestinal gas, whereas others are gas-utilizing and decrease gas accumulation. *Bifidobacterium* and *Lactobacillus* species can competitively inhibit gas-producing microbes and metabolize dietary substrates (e.g., fiber) to reduce gas production [4,5]. Additionally, commensal microbes (e.g., *Blautia* spp.) can utilize intestinal gases and convert them into beneficial short-chain fatty acids (SCFA) [6]. Second, slow GI motility contributes to bloating and constipation, and certain microbes produce metabolites that regulate motility. For example, butyrate-producing microbes stimulate colonic contractions via the Src signaling pathway [7,8]. Third, visceral hypersensitivity, discomfort experienced at a lower threshold than normal, is an important mechanism underlying bothersome bloating symptoms, with the gut microbiome playing a key role. Clinically, disruption of GI barrier function is linked to increased sensitivity to visceral and somatic stimuli [9]. Mechanistically, specific microbes improve barrier function by promoting tight junction assembly through SCFA production and by stimulating mucin synthesis via microbiome-mediated production of urolithin A (UroA) [10,11].

Probiotics are live microorganisms that confer health benefits to the host and have been extensively studied in irritable bowel syndrome (IBS) through randomized, placebo-controlled trials [12]. While such studies are valuable in patients with IBS, it is equally important to assess their effects on bloating, gas, and abdominal discomfort in generally healthy populations without a medical diagnosis. Findings in clinician diagnosed patient populations may not translate to otherwise healthy individuals, who typically experience milder symptoms, do not seek medical care, and often self-manage their GI discomfort. Previous probiotic studies in otherwise healthy individuals have not demonstrated benefits for bloating and gas-related symptoms. A trial of a single-strain probiotic (*B. infantis* 35624) over 4 weeks found no improvement in bloating or abdominal discomfort compared with placebo [13]. Likewise, a 3-week trial of a two-strain probiotic (*L. acidophilus* Rosell-52 and *B. longum* Rosell-175) failed to demonstrate benefit for bloating or gas-related symptoms [14]. Lastly, a clinical trial in obese adults did not demonstrate any benefit in reducing bloating from *Bifidobacterium*-based interventions [15].

Synbiotics may exert greater biological effects than probiotics alone by combining live microorganisms with prebiotics, dietary substrates that selectively promote the growth or function of beneficial microbes. Probiotic microbes formulated with a polyphenol-based prebiotic have shown mechanistic and preclinical evidence supporting potential benefits for bloating and other GI symptoms [16]. Preclinically, this combination increased butyrate production, which may modulate GI motility and improve visceral hypersensitivity, both implicated in bloating [17]. Pomegranate extract, the prebiotic component of the synbiotic, is a rich source of ellagitannins. The biological effect of these polyphenols is driven by a two-step biotransformation by certain gut microbes. Specifically, the synbiotic includes *Lactobacillus* spp. (*L. rhamnosus*, *L. plantarum*, *L. casei*), which metabolize ellagitannins into a key precursor, ellagic acid. This precursor is then metabolized by resident microbes to produce UroA. UroA is a key metabolite that maintains intestinal barrier integrity, supports mucin production, decreases inflammation in the gut, and may mitigate visceral hypersensitivity implicated in bloating.

In a randomized, placebo-controlled trial in generally healthy individuals, these synbiotic components increased beneficial microbes and microbiome-derived metabolites linked to improved GI symptoms [16]. These biological changes map to mechanisms relevant to bloating, including intestinal gas, GI motility, and visceral hypersensitivity. Supplementation significantly increased the alpha-diversity of *Bifidobacterium* and *Lactobacillus* species both rapidly (after one week) and persistently (after three months) compared to placebo. Specific gas-reducing microbes also increased, including *B. breve* SD-BR3-IT, *L. plantarum* SD-LP1-IT, and native taxa such as *Blautia*. This composition further increased butyrate-producing genera (*Roseburia*, *Butyribacter*), elevating intestinal butyrate, which stimulates colonic contraction and reduces bloating and constipation. Lastly, supplementation increased UroA levels and the abundance of butyrate-producing microbes, which may contribute to enhanced gut barrier function through stimulation of mucin synthesis and modulation of tight junction protein expression. Improved barrier function may, in turn, mitigate visceral hypersensitivity, a key driver of bloating.

This prior clinical trial did not assess clinical GI symptoms, as it was focused on mechanistic outcomes. Accordingly, we conducted a randomized, placebo-controlled trial to evaluate the health benefits of a multi-species synbiotic, with a focus on GI symptoms including bloating, in a diverse, generally healthy, non-clinical population.

## 2. Methods

### 2.1. Clinical Trial Design

A randomized, double-blind, placebo-controlled, parallel-group, decentralized clinical trial was conducted among generally healthy adults (Radicle Science, Inc., Encinitas, CA, USA). The study was approved by an independent Institutional Review Board (Sterling IRB, Atlanta, GA, USA; IRB #11062, 15 August 2023) and conducted in accordance with the Declaration of Helsinki. All participants signed written consent forms to participate in this study. Participants were randomized 1:1 and administered daily capsules of a multi-species synbiotic or a matching placebo for 6 weeks. Randomization allocation sequence was electronically generated using MS XLS (Redmond, WA, USA) and randomization implemented with the Qualtrics XM platform (Provo, UT, USA). Key outcomes included validated measures of GI quality-of-life, bloating and gas, and abdominal pain/discomfort. Additional endpoints assessed constipation, regularity, mood, and safety. With the assumptions of 90% power to find a difference between study arms with a two-sided *p*-value of 0.05, approximately 210 participants were needed. Given anticipated attrition rates from previous decentralized studies, approximately 350 participants allowed an adequate sample size. Trial reporting follows CONSORT guidelines (Supplement File S1).

### 2.2. Study Participants

Participants for the decentralized trial were recruited online from across the United States through social media advertisements, electronic mailing lists, and a third-party consumer network with nationwide representation. Informed consent was obtained electronically, and participants received a digital copy of the signed consent form. All data were collected via online questionnaires accessed through participant-specific hyperlinks provided at study time points.

The study population included otherwise healthy male and female adults with self-reported bloating/indigestion for ≥3 months who identified reduced bloating/indigestion as their primary goal. Exclusion criteria included pregnancy or breastfeeding; liver, kidney, or heart disease (NYHA class III/IV congestive heart failure, atrial fibrillation, uncontrolled arrhythmia); heavy alcohol consumption (≥3 drinks/day); current or recent (≤3 months) chemotherapy or immunotherapy; medications with substantial safety risk (e.g., anticoagulants, antihypertensives, anxiolytics, antidepressants, corticosteroids, antipsychotics, MAOIs, thyroid products); or known moderate-to-severe interactions with active ingredients (e.g., antibiotics, antifungals, antivirals). Additional exclusions included concurrent enrollment in another clinical trial and operational limitations (lack of reliable daily internet access, inability to read English, or inability to provide a valid U.S. shipping address). Participants completed a validated baseline dietary record (Starting the Conversation: Diet) [18]. Additionally, participants were instructed to avoid changing their diet through the duration of the trial.

**Figure 1:**
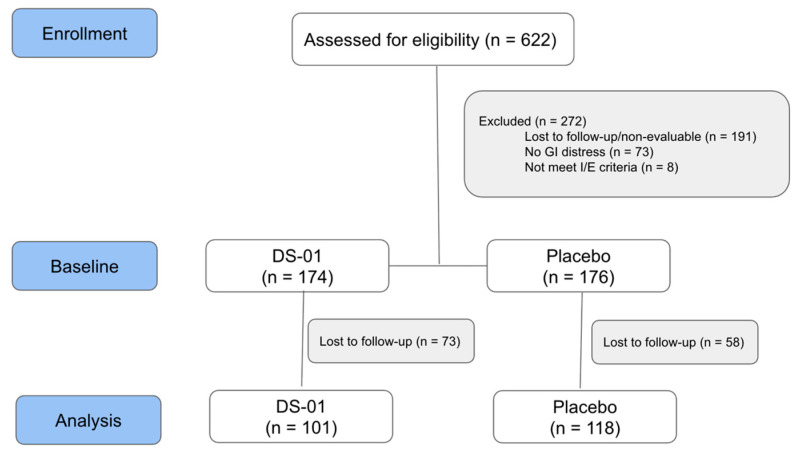
Study Flow Diagram: Participant flow through the decentralized trial.

Figure 1 shows the CONSORT Flow Diagram. A total of 622 individuals were assessed for eligibility. Evaluable data was available for 350 participants at baseline and 219 participants at Week 6.

### 2.3. Multi-Species Synbiotic Intervention

Participants were randomized to ingest a multi-species synbiotic with capsule-in-capsule delivery technology (ViaCap) totaling 400 mg of prebiotic Indian pomegranate (*Punica granatum*, >40% polyphenols) extract and 53.6 billion AFU of a multi-species probiotic containing 24 different bacteria strains: *Bifidobacterium longum* SD-BB536-JP, *Bifidobacterium breve* SD-BR3-IT, *Lactiplantibacillus plantarum* SD-LP1-IT, *Lacticaseibacillus rhamnosus* SD-LR6-IT, *Lacticaseibacillus rhamnosus* HRVD113-US, *Bifidobacterium infantis* SD-M63-JP, *Bifidobacterium lactis* SD-BS5-IT, *Bifidobacterium lactis* HRVD524-US, *Lactobacillus crispatus* SD-LCR01-IT, *Lacticaseibacillus casei* HRVD300-US, *Bifidobacterium breve* HRVD521-US, *Bifidobacterium longum* HRVD90b-US, *Bifidobacterium lactis* SD150-BE, *Limosilactobacillus fermentum* SD-LF8-IT, *Lacticaseibacillus rhamnosus* SD-GG-BE, *Limosilactobacillus reuteri* RD830-FR, *Ligilactobacillus salivarius* SD-LS1-IT, *Bifidobacterium lactis* SD-CECT8145-SP, *Bifidobacterium longum* SD-CECT7347-SP, *Lacticaseibacillus casei* SD-CECT9104-SP, *Lactiplantibacillus plantarum* SD-LPLDL-UK, *Bifidobacterium lactis* SD-MB2409-IT, *Bifidobacterium adolescentis* SD-BA5-IT, and *Limosilactobacillus reuteri* SD-LRE2-IT (DS-01, Seed Health, Inc., Venice, CA, USA) or a matching placebo (rice flour). The multi-species synbiotic included strains that have shown benefits for several GI symptoms [19]. Participants were instructed to ingest 1 capsule once per day for the first 3 days followed by 2 capsules per day for the remainder of the study. The product was administered once daily in the morning on an empty stomach, immediately before breakfast, starting at baseline (Day 0) and continuing for 6 weeks.

### 2.4. Outcomes: Efficacy and Safety

The primary outcome was gastrointestinal quality of life at Week 6, assessed with the Digestion-associated Quality-of-Life Questionnaire (DQLQ), a validated tool for otherwise healthy individuals [20]. Secondary clinical outcomes included bloating and gas (NIH PROMIS-GI 13a) and abdominal pain/discomfort (NIH PROMIS-GI 5a), both validated in general populations [21]. Anxiety (NIH PROMIS-Anxiety 4a) and safety were also assessed. Other mood-related symptoms evaluated included depression (NIH PROMIS-Anxiety 4A) and libido (NIH PROMIS Sexual Interest). Lower gastrointestinal outcomes were evaluated as follows: constipation was measured on a 5-point Likert scale (1 = no discomfort, 5 = very significant discomfort) in response to the question: “Have you experienced discomfort due to constipation? (Constipation is a reduced ability to empty your bowels and/or difficulty having a bowel movement).” Regularity was assessed on a 5-point Likert scale (1 = not at all, 5 = significantly) in response to the question: “To what extent do you feel the study product has improved the regularity and quality of your bowel movements?” Adverse events and their severity were monitored. Severity was graded based on the utilization of medical services in response to the event, following the Common Terminology Criteria for Adverse Events (CTCAE v5.0, USDHHS).

### 2.5. Statistical Analysis

Baseline demographic differences between study arms were assessed using Fisher’s Exact Test or t-tests for each category (age, race, ethnicity, education level, sex at birth, BMI). Study endpoints at each time point were analyzed using the non-parametric Mann–Whitney U (MWU; Wilcoxon Rank Sum) test to compare the active and placebo arms. Safety data were evaluated with Fisher’s Exact Test to compare adverse events. Symptom-specific subpopulations were examined using Fisher’s Exact Test to assess improvement over time between arms. Participants were included if they had at least one week of adherent data beyond baseline, defined as reporting product use on the majority of days in a given week. Missing data were handled using a conservative imputation method (last observation carried forward; applied only to the final two time points). Statistical significance was set a priori at a two-tailed alpha of 0.05. Descriptive statistics were generated for all demographic, safety, and endpoint measures. Continuous variables were summarized using mean ± standard deviation or median with interquartile range, as appropriate, while categorical variables were reported as frequencies and percentages. All analyses were conducted in R (version 4.4.3) using the tidyverse (v2.0.0) for data cleaning and summarization and rstatix (v0.7.2) for Mann–Whitney U and Fisher’s Exact Tests.

## 3. Results

### 3.1. Participant Demographics

Table 1 shows participant demographics at baseline. Overall, participants from 40 U.S. states and 1 U.S. territory were represented in the clinical trial, with the trial population distribution generally mirroring US Census population data.

### 3.2. Gastrointestinal Quality-of-Life

**Figure 2:**
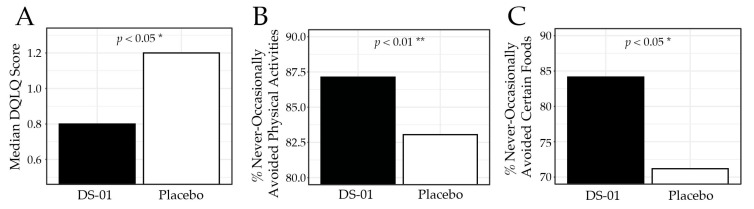
Digestion-associated Quality-of-Life Questionnaire (DQLQ) at Week 6. (**A**) median DQLQ composite score by study arm, (**B**) percent (%) of participants who responded “never”, “rarely”, or “occasionally” to the DQLQ question “Due to digestive events and experiences in the past 7 days,—Physical activities (running, walking, gardening, golfing, etc.) were unpleasant or avoided.”, (**C**) percent of participants who responded “never”, “rarely”, or “occasionally” to the DQLQ question “Due to digestive events and experiences in the past 7 days,—I avoided certain foods.” (* *p* < 0.05, ** *p* < 0.01).

The primary outcome, gastrointestinal quality-of-life measured by validated DQLQ (where a lower score is a better outcome), was significantly improved in the synbiotic arm compared with placebo at Week 6 (median score 0.80 vs. 1.20; *p* < 0.05; Figure 2), corresponding to an 81.4% improvement from baseline compared with 72.1% in the placebo arm. Specifically, participants in the synbiotic arm reported digestive events as less impactful on physical activities (87.1% vs. 83.1%; *p* < 0.01), less likely to result in food avoidance (84.2% vs. 71.2%; *p* < 0.05), and associated with greater food enjoyment (85.2% vs. 69.5%; *p* < 0.05). Table 2 presents the DQLQ results for each study week.

### 3.3. Bloating and Gas

**Figure 3:**
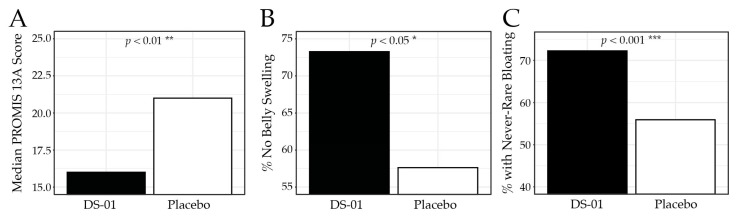
PROMIS-GI 13a Bloating and Gas results at Week 6. (**A**) median PROMIS-GI 13a composite score by study arm, (**B**) percent (%) of participants who responded “no”, to the PROMIS-GI 13a question “In the past 7 days, did you have swelling in your belly?”, (**C**) percent of participants who responded “never”, or “rarely” to the PROMIS-GI 13a question “In the past 7 days, how often did you feel bloated?” (* *p* < 0.05, ** *p* < 0.01, *** *p* < 0.001).

Bloating and gas were assessed using the validated PROMIS-GI 13a, where lower scores indicate fewer symptoms. At Week 6, participants in the synbiotic arm reported significantly less bloating and gas than placebo (median 16.0 vs. 21.0; *p* < 0.01; Figure 3), corresponding to a 54.3% improvement from baseline compared with 42.5% in the placebo arm. Specifically, 73.3% of participants in the synbiotic arm reported no belly swelling versus 57.6% in the placebo arm (*p* < 0.05), and 72.3% reported never/rarely experiencing bloating compared with 55.9% in the placebo arm (*p* < 0.001). Table 3 presents the PROMIS-GI 13a results for each study week.

### 3.4. Abdominal Discomfort/Pain

**Figure 4:**
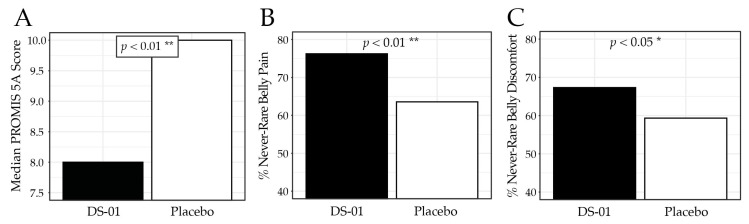
PROMIS-GI 5a Abdominal Discomfort/Pain results at Week 6. (**A**) median PROMIS-GI 5a composite score by study arm, (**B**) percent (%) of participants who responded “never” or “one day”, to the PROMIS-GI 5a question “In the past 7 days, how often did you have belly pain?” (**C**) percent of participants who responded “never”, or “rarely” to the PROMIS-GI 5a question “In the past 7 days, how often did you have discomfort in your belly?” (* *p* < 0.05, ** *p* < 0.01).

Abdominal discomfort and pain were assessed using the PROMIS-GI 5a, where lower scores indicate fewer symptoms. At Week 6, the synbiotic arm reported significantly less discomfort than placebo (median 8.0 vs. 10.0; *p* < 0.01; Figure 4), corresponding to a 46.7% improvement from baseline compared with 37.5% in the placebo arm. Specifically, participants receiving the synbiotic reported less frequent abdominal pain (never/one day per week): 76.2% vs. 63.6%; *p* < 0.01) and abdominal discomfort (never/rarely: 67.3% vs. 59.3%; *p* < 0.05). Table 4 presents the PROMIS-GI 5a results for each study week.

### 3.5. Constipation and Regularity

**Figure 5:**
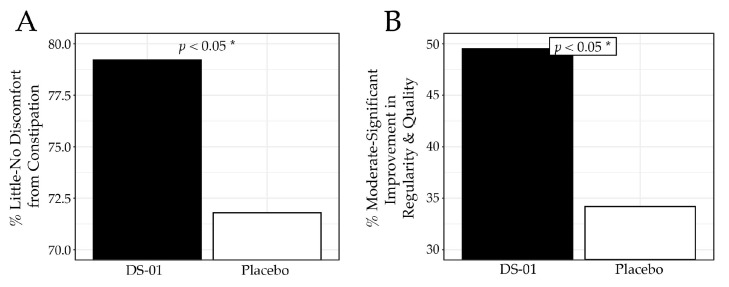
Constipation and Regularity results at Week 6. (**A**) percent (%) of participants who responded “no discomfort” or “slight discomfort” to the question, “Have you experienced discomfort due to constipation? (Constipation is a reduced ability to empty your bowels and/or difficulty having a bowel movement)”, (**B**) percent of participants who responded “moderately”, “noticeably” or “significantly” to the question, “To what extent do you feel the study product has improved the regularity and quality of your bowel movements?” (* *p* < 0.05).

Overall, the synbiotic improved both constipation and bowel regularity. At Week 6, participants in the synbiotic arm reported less frequent constipation-related discomfort (no/slight: 79.2% vs. 71.8%; *p* < 0.05; Figure 5A) and greater improvement in regularity and quality of bowel movements (moderately/noticeably/significantly: 49.5% vs. 34.2%; *p* < 0.05; Figure 5B) compared with placebo. Further analysis showed that in a clinically meaningful subgroup with baseline constipation, there was improvement in lower GI symptoms in the synbiotic arm compared to placebo. Specifically, the group receiving the synbiotic reported no/slight constipation-related discomfort in 75.6% of participants in contrast to only 67.7% in the placebo (*p* < 0.05), while improvements in bowel movement regularity/quality rose to 52.6% in the synbiotic arm compared to 33.3% in the placebo arm (*p* < 0.05) at Week 6.

### 3.6. Mood

No significant difference in anxiety was observed with the PROMIS-Anxiety 4a in the overall population. However, in a clinically relevant subgroup with mild anxiety, the synbiotic demonstrated a beneficial effect on an anxiety-related symptom (“hard to focus on anything other than my anxiety”) at Week 6. A significantly greater percentage of participants in the synbiotic arm reported a noticeable improvement (at least a 1-point reduction on the 5-point Likert scale) compared to placebo: 66.0% vs. 45.5% (*p* < 0.05). No significant difference was observed in depression or libido in the overall population.

### 3.7. Safety

Overall, the multi-species synbiotic was well-tolerated, with no serious adverse events reported. Safety was prospectively monitored throughout the trial, and there were no significant differences observed between arms (*p* > 0.05), including total adverse events, and symptom-specific events including dizziness, drowsiness, dry mouth, headache, insomnia, nausea, numbness/tingling, and other GI symptoms (Supplement Tables S1–S11).

## 4. Discussion

This randomized, placebo-controlled trial demonstrates the efficacy of a multi-species synbiotic in alleviating GI symptoms. This study represents the largest probiotic/synbiotic clinical trial evaluating bloating to include both men and women, as well as the largest in a non-patient population. The methodology was further strengthened by the use of validated GI endpoints and real-world demographic diversity. A previous trial evaluating *Bifidobacterium infantis* 35624 for similar symptoms was larger (*n* = 362) but had meaningful limitations [22], as eligibility was restricted to women with IBS, a clinical population, which limits the generalizability of those findings to men and otherwise healthy individuals. Additionally, this large dose-finding trial demonstrated a paradoxical dose-dependent effect, where only the middle dose (10^8^ CFU) showed improvement in abdominal discomfort/pain and bloating, but interestingly not the high dose [22]. Importantly, a subsequent large clinical trial by Ringel-Kulka and colleagues with the same probiotic strain in generally healthy participants showed no benefit for bloating or abdominal discomfort [13]. These findings underscore the difficulty of detecting beneficial effects in diverse, non-patient populations with less severe symptoms, a challenge successfully addressed in the present study. Overall, several key findings emerged from this randomized, placebo-controlled trial.

First, the multi-species synbiotic improved GI quality of life compared with placebo as measured by the DQLQ. This broad digestion-associated tool is validated in otherwise healthy adults, and is a meaningful metric for individuals with GI symptoms. Notably, prior probiotic/synbiotic studies have generally not demonstrated a significant improvement for this outcome. For example, a multi-ingredient green powder enriched with *L. acidophilus* and *B. bifidum* failed to improve DQLQ scores in healthy adults [23]. In the present study, improvements were largely driven by digestive events no longer interfering with physical activity or causing food avoidance, findings consistent with real-world experience, where bothersome GI symptoms often reduce physical activity and restrict diet. Interestingly, participants in the synbiotic arm also reported greater food enjoyment, which may be linked directly to less food avoidance or possibly modulation of food reward pathways reported along the gut–brain axis [24].

Second, the multi-species synbiotic significantly improved bloating and gas compared with placebo, as measured by the validated PROMIS-GI 13a. The effect was meaningful: 72.3% of participants in the synbiotic arm reported never or rarely experiencing bloating versus 55.9% in the placebo group (*p* < 0.001). These findings highlight a notable improvement in digestive comfort in an otherwise healthy population, addressing an important gap in digestive health research. This is particularly important given the lack of evidence from prior probiotic or synbiotic interventions in similar populations. For example, a clinical trial evaluating *Bacillus coagulans* GBI-30, 6086 for postprandial GI symptoms showed no benefit for bloating on the Severity of Dyspepsia Assessment (SODA) or Gastrointestinal Symptom Rating Scale (GSRS) [25]. Likewise, other randomized controlled trials of full-formulation probiotics have failed to demonstrate improvements in bloating among generally healthy individuals [13,14].

Third, the multi-species synbiotic significantly improved abdominal discomfort/pain, as measured by the validated PROMIS-GI 5a. The effect was meaningful, with 67.3% of participants in the synbiotic arm reporting never or rarely experiencing abdominal discomfort versus 59.3% in the placebo arm (*p* < 0.05). This outcome aligns with improvements in bloating, as abdominal discomfort can be closely associated with bloating severity. Mechanistically, these effects may relate to reduced visceral hypersensitivity. The polyphenol-rich pomegranate extract contained in the synbiotic may drive production of UroA, a metabolite shown to support intestinal barrier integrity, mucus production, and modulate pathways that mitigate visceral hypersensitivity. Additionally, clinical evidence shows that butyrate can decrease visceral hypersensitivity in healthy populations [26], and a recent trial demonstrated that a synbiotic increased butyrate-producing microbes such as *Roseburia* and *Butyribacter* [16].

Fourth, the multi-species synbiotic improved lower GI symptoms, including constipation and regularity. This finding is notable because most probiotic studies assessing bowel habits have focused on patients with medically diagnosed functional constipation or IBS-C defined by ROME criteria [27,28,29,30]. In contrast, evaluation of constipation in otherwise healthy individuals is uncommon due to challenges in endpoint sensitivity, yet it is more generalizable. Despite enrolling participants primarily with self-reported upper GI symptoms such as bloating, this trial demonstrated that the multi-species synbiotic significantly improved constipation and regularity symptoms.

Fifth, the multi-species synbiotic did not significantly improve anxiety in the overall study population, which was expected given the low baseline anxiety levels typical of a broad GI cohort. However, subgroup analysis showed improvement among individuals with self-reported mild anxiety. Although further research is needed, SCFAs may contribute to this effect, as butyrate administration was linked with improved emotional scores in a recent clinical trial [31]. Future trials evaluating this synbiotic in populations with self-reported anxiety may therefore be warranted.

Sixth, the multi-species synbiotic was well-tolerated, with no serious adverse events reported. This finding is consistent with a previous clinical trial that also demonstrated safety based on vital signs and clinical chemistry markers, including renal and liver function [16].

This study has several limitations. Although the decentralized design enhanced recruitment and generalizability, it resulted in an increased dropout rate, and conservative analytical methods were applied. In particular, geographic diversity and potential associated differences in dietary patterns introduce natural variability that may attenuate effect sizes; however, this heterogeneity also enhances external validity by reflecting the real-world population of individuals who experience bloating and GI symptoms. Additionally, we did not conduct biomarker and microbiome assessments in this trial. Mechanistic effects of these synbiotic components on gut microbiome composition, Urolithin A, and butyrate have been characterized in a separate randomized, placebo-controlled study [16]. However, these measures were not repeated in this trial, potentially limiting direct mechanistic conclusions. Furthermore, the trial relied upon validated self-reported instruments rather than objective physician-administered measurements (e.g., direct measurement of abdominal girth). While this design choice may have contributed to the response rate in the placebo arm, it reflects the subjective nature of bloating that most individuals experience. Similarly, regarding the population, the decentralized design prioritized external validity over clinical evaluation, reflecting the reality that bloating is a prevalent, often self-managed condition. As such, participants did not have a physician evaluation at baseline (e.g., clinically validated medical history and physical examination). We acknowledge this limits the formal identification of physician-verified severe bloating conditions (e.g., objective visible distension) and may result in the inclusion of underdiagnosed functional bowel conditions. However, this heterogeneity is intentional and strengthens generalizability among a real-world population seeking relief from GI distress. Finally, regarding participant classification, although individuals reported intermittent bloating/indigestion, they did not report a medical diagnosis of a functional GI disorder (e.g., meeting ROME IV criteria). Thus, consistent with prior research in generally healthy populations, they were classified as otherwise healthy adults for the purposes of this clinical trial. We acknowledge, however, that self-reported GI symptoms, while common in the general public, may technically fall outside the strictest interpretation of healthy (e.g., WHO definition). Despite these limitations, to our knowledge, this is the largest probiotic/synbiotic clinical trial evaluating bloating and gas symptoms to include both men and women, the largest in a non-patient population, and the first synbiotic to show meaningful improvements in bloating and gas in a diverse, generally healthy population.

## 5. Conclusions

This randomized, placebo-controlled trial in otherwise healthy adults with self-reported bloating/indigestion suggests daily supplementation with a multi-species synbiotic improved GI quality-of-life, bloating and gas, and abdominal discomfort/pain based on validated outcome measures. The multi-strain synbiotic also improved constipation and bowel regularity symptoms within this population. Overall, these findings support the potential role of a multi-species synbiotic in mitigating a range of GI symptoms in this diverse, real-world, non-patient population.

## Figures and Tables

**Table 1:** Baseline Demographics.

Measure	DS-01 (*n* = 174)		Placebo (*n* = 176)		
	Mean/N	SD/%	Mean/N	SD/%	*p*-Value
Age (years)	45.2	12.2	45.1	11.7	0.90
Sex (% Male)	56	32.2%	57	32.4%	1.00
BMI (kg/m^2^)	29.4	7.8	29.2	8.4	0.78
Race					0.088
American Indian/Alaska Native	0	0.0%	5	2.8%	
Asian	14	8.0%	8	4.5%	
Black	24	13.8%	22	12.5%	
Multi-racial	15	8.6%	18	10.2%	
Native Hawaiian/Pacific Islander	1	0.6%	2	1.1%	
White	117	67.2%	110	62.5%	
Some other race	1	0.6%	5	2.8%	
Prefer not to say	2	1.1%	6	3.4%	
Hispanic, LatinX, or Spanish	20	11.5%	29	16.5%	0.38
Education					0.34
Less than high school	0	0.0%	1	0.6%	
High school diploma	27	15.5%	17	9.7%	
Some college, no degree	30	17.2%	43	24.4%	
Bachelors or associate degree	78	44.8%	81	46.0%	
Masters or professional degree	26	14.9%	23	13.1%	
Trade/technical/vocational degree	12	6.9%	9	5.1%	
Prefer not to say	1	0.6%	2	1.1%	
DQLQ	4.5	1.9	4.5	1.9	0.98
PROMIS 13A Bloating and Gas	34.9	11.7	36.0	10.9	0.34
PROMIS 5A Belly Pain	15.7	4.5	16.1	4.7	0.45

**Table 2:** Digestion-associated Quality-of-Life Questionnaire (DQLQ) at each study week.

Study Week	Median			*p*-Value
	DS-01	Placebo	Difference	
Baseline	4.30	4.30	0.00	0.928
Week 1	1.40	1.75	−0.35	0.547
Week 2	1.30	1.40	−0.10	0.759
Week 3	1.00	1.40	−0.40	<0.05 *
Week 4	0.90	1.20	−0.30	<0.05 *
Week 5	0.85	1.10	−0.25	0.077
Week 6	0.80	1.20	−0.40	<0.05 *

* *p* < 0.05.

**Table 3:** PROMIS-GI 13a Bloating and Gas Score at each study week.

Study Week	Median			*p*-Value
	DS-01	Placebo	Difference	
Baseline	35.0	36.5	−1.5	0.415
Week 1	22.0	24.5	−2.5	0.143
Week 2	20.0	20.0	0.0	0.432
Week 3	19.0	19.0	0.0	0.254
Week 4	16.0	22.0	−6.0	<0.001 ***
Week 5	18.0	21.0	−3.0	<0.05 *
Week 6	16.0	21.0	−5.0	<0.01 **

* *p* < 0.05, ** *p* < 0.01, *** *p* < 0.001.

**Table 4:** PROMIS-GI 5a Abdominal Discomfort/Pain Score at each study week.

Study Week	Median			*p*-Value
	DS-01	Placebo	Difference	
Baseline	15.0	16.0	−1.0	0.444
Week 1	10.0	12.0	−2.0	0.148
Week 2	10.0	11.0	−1.0	0.121
Week 3	9.0	11.0	−2.0	<0.01 **
Week 4	8.0	11.0	−3.0	<0.05 *
Week 5	9.0	10.0	−1.0	0.089
Week 6	8.0	10.0	−2.0	<0.01 **

* *p* < 0.05, ** *p* < 0.01.

## Data Availability

Data and statistical analyses are available for non-commercial scientific inquiry and/or educational purposes if request and use does not violate IRB restrictions and/or research agreement terms.

## Supplementary Materials

The following supporting information can be downloaded at: https://www.mdpi.com/article/10.3390/nu18020255/s1; Supplement File S1: CONSORT 2025 checklist. Table S1: Participants who reported at least one AE; Table S2: Participants who reported dizziness by study week; Table S3: Participants who reported drowsiness by study week; Table S4: Participants who reported nausea by study week; Table S5: Participants who reported upset stomach/cramping by study week; Table S6: Participants who reported constipation by study week; Table S7: Participants who reported diarrhea by study week; Table S8: Participants who reported dry mouth by study week; Table S9: Participants who reported headache by study week; Table S10: Participants who reported insomnia by study week; Table S11: Participants who reported tingling or numbness by study week. Reference [32] is cited in the Supplementary Materials.
